# A Successive Approximation Time-to-Digital Converter with Single Set of Delay Lines for Time Interval Measurements

**DOI:** 10.3390/s19051109

**Published:** 2019-03-05

**Authors:** Jakub Szyduczyński, Dariusz Kościelnik, Marek Miśkowicz

**Affiliations:** 1 Department of Electronics, AGH University of Science and Technology, 30-059 Kraków, Poland; koscieln@agh.edu.pl; 2 Department of Measurement and Electronics, AGH University of Science and Technology, 30-059 Kraków, Poland; miskow@agh.edu.pl

**Keywords:** successive approximation, time-to-digital converter (TDC), feedforward architecture, time interval measurement

## Abstract

The paper is focused on design of time-to-digital converters based on successive approximation (SA-TDCs—Successive Approximation TDCs) using binary-scaled delay lines in the feedforward architecture. The aim of the paper is to provide a tutorial on successive approximation TDCs (SA-TDCs) on the one hand, and to make the contribution to optimization of SA-TDC design on the other. The proposed design optimization consists essentially in reduction of circuit complexity and die area, as well as in improving converter performance. The main paper contribution is the concept of reducing SA-TDC complexity by removing one of two sets of delay lines in the feedforward architecture at the price of simple output decoding. For 12 bits of resolution, the complexity reduction is close to 50%. Furthermore, the paper presents the implementation of 8-bit SA-TDC in 180 nm CMOS technology with a quantization step 25 ps obtained by asymmetrical design of pair of inverters and symmetrized multiplexer control.

## 1. Introduction

Design of modern integrated circuit is driven mainly by downscaling of complementary metal oxide semiconductor (CMOS) technology. Digital electronics fully benefit from reduced transistor geometry in terms of die area, power per functionality, and switching speed. On the other hand, the design of analog and mixed-signal circuits becomes more and more challenging because a reduction of transistor dimensions implies a decrease of the supply voltage. While older CMOS technologies utilized high supply voltages (from 15 V to 2.5 V), below the 100 nm technology feature size, the maximum operating voltage is near or below 1 V. This makes the fine quantization of the amplitude increasingly difficult. Furthermore, according to the fundamental law of MOS physics, the intrinsic gain of a single MOS transistor (*g_m_*/*g_ds_*) decreases with lowering the supply voltage [1,2].

**Figure 1:**
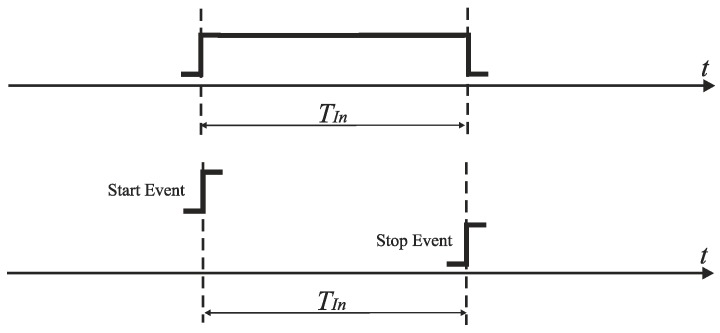
Encoding information in time domain in the form of input time interval *T_In_*.

Due to the sharp increase of switching speed and the continuous reduction of voltage headroom in deep-submicron CMOS technologies, the resolution of encoding signals in the time domain becomes superior to the resolution of analog signal amplitude in the voltage domain [3]. The technique of encoding signals in time instead of in amplitude is expected to be further improved by advances in CMOS technology. To neutralize the scaling-induced design challenges, the signals that originate in the amplitude domain (e.g., voltage) are proposed to be encoded in the time domain [3,4]. In *time-mode signal processing*, information is represented by the time intervals between discrete events (Figure 1) rather than by the voltages, or currents in electric networks. The detrimental effect of technology scaling on the performance of voltage or current-mode analog signal processing is in time-mode circuits alleviated. Furthermore, time-mode circuits are less sensitive to interferences than voltage-mode circuits (e.g., cross talk, or substrate coupling).

Encoding signals in time is in fact not new and has been used for example in multislope analog-to-digital converters. The very first approach of trading voltage resolution against time resolution is the Sigma-Delta modulator (SDM). In the SDM, a coarse quantizer causes a considerable quantization error that is balanced by oversampling with noise shaping [2]. An extreme implementation of this concept leads to encoding any signal information in the time domain.

One of major inspirations to represent signals based on timing instants comes from neuroscience. The examples of biologically-inspired time encoding techniques are spiking neurons, where the information is conveyed by the spike firing time. In the wake of brain’s efficient data-driven communication, the neuromorphic electronic systems are designed to sense, communicate, compute, and learn using asynchronous event-based communication [5]. Time encoding of events is associated in general with *event-based signal processing*, an emerging research area that consists in representing signals by a sequence of discrete events (e.g., by level-crossing sampling) rather than by periodic samples [6,7].

Time-mode circuits are essentially designed as digital because digital circuits by definition are unable to resolve any information in the amplitude domain while they have a high resolution in the time domain. Encoding signals in time at early stage of signal processing chain enables moving most system components to the digital domain. The digital nature of time-mode circuits allows them to be migrated from one generation of technology to another with the minimum design overhead. An example of migration of analog design complexity to the digital domain using the time-mode approaches is a development of *digital RF*, which transforms the functionality of radio-frequency (RF) front-end electronics into digitally intensive mixed-signal realizations using scaled CMOS technology [8]. The use of time-to-digital converter as a replacement of the conventional phase/frequency detector and charge pump in all-digital phase-locked loops (ADPLLs) allows in PLLs to replace the loop filter requiring large and leaky integrating capacitors by a simple digital filter [9].

Time-to-digital converters are devices that convert time domain information into a digital representation [1,2,3,4,10,11,12,13], so they act as analog-to-digital converters for time-mode signal processing systems. Thus, TDCs are an enabler for the time-domain digital processing of continuous signals. The TDCs have been originally developed for precise time interval measurements in space science and high-energy physics in the 80 s [13,14]. With the improved time resolution TDCs have been widely used in time-of-flight measurement applications [15,16,17,18,19,20], for example in laser range finders [1,2,12].

Nowadays, the time-to-digital converters find a broad spectrum of applications such as digital storage oscillators, laser-based vehicle navigation systems, medical imaging and instrumentation, infinite and finite impulse response filters, all digital phase-locked loops, clock data recovery, and channel select filters for software-defined radio [1,2,10,12,13]. In consumer electronics, the first common application of TDC was its use as a phase detector in ADPLLs [3,9,11]. This application has triggered extensive research on TDCs, especially in the field of on-chip frequency synthesizers, and resulted in a development of new conversion algorithms, architectures, and implementations to improve their performance in terms of time resolution, conversion speed, and power [12]. Various types of TDC architectures have been proposed to address these objectives. By analogy to analog-to-digital converters, they can be classified into Nyquist-rate TDCs and oversampled TDCs [16]. Nyquist-rate TDCs include counter TDCs, delay line and Vernier line TDCs [10,12,21], TDCs with interpolation, pulse-shrinking or pulsestretching, successive approximation TDCs [22,23,24,25,26,27,28,29,30,31,32,33,34,35,36,37], flash and pipelined TDCs [1,2]. Noise-shaping TDCs are aimed to suppress the quantization noise using system-level techniques such as Sigma-Delta modulation by moving most of in-band quantization noise outside the signal band in order to achieve a large signal-to-noise ratio (SNR) and improve effective TDC resolution. Noise-shaping TDCs include gated ring oscillator TDCs [38], switched ring oscillator TDCs, MASH TDCs [39], Sigma-Delta TDCs [40,41], and their combinations [1,2].

This paper focuses on time-to-digital converters based on successive approximation (SA-TDCs—Successive Approximation TDCs) [22,23,24,25,26,27,28,29,30,31,32,33,34,35,36,37]. The aim of the paper is to give a tutorial on successive approximation TDCs (SA-TDCs) on one hand, and to make the contribution to optimization of SA-TDC design on the other. The main paper contribution is to minimize SA-TDC complexity and die area by removing one of two sets of delay lines in the feedforward architecture at the price of simple output decoding. Furthermore, the improvement of converter performance in the exemplified implementation of 8-bit SA-TDC in 180 nm CMOS technology by asymmetrical design of pair of inverters and symmetrized multiplexer control is reported. The careful analysis shows that the reduction of complexity for 8-bit SA-TDC is around 20–30%, while for 12 bits respectively almost 50%. The present study is an extension of the conference papers [22,23,24].

The paper is organized as follows: the first part (Section 2, Section 3, Section 4 and Section 5) is intended to be a tutorial on SA-TDCs. In Section 2, a brief overview of representative TDC methods is outlined. Section 3 and Section 4 summarize successive approximation algorithms adopted to analog-to-digital conversion, and the operation principle of the TDC based on monotone successive approximation. Furthermore, in Section 5, the basic model of SA-TDC in the feedforward architecture and its variants are introduced. The second part of the paper (Section 6, Section 7 and Section 8) reports the contribution to optimization of SA-TDC design. In particular, Section 6 discusses an approach to minimize SA-TDC complexity by removing one of two sets of delay lines in the feedforward architecture at the price of simple output decoding. Section 7 presents the implementation details of 8-bit SA-TDC in 180 nm CMOS technology with a quantization step 25 ps obtained by asymmetrical design of pair of inverters, and a method to reduce INL and DNL converter nonlinearities by symmetrized multiplexer design. Finally, the analysis of device mismatch and time jitter, as well as the impact of temperature and supply voltage variations on the performance of implemented TDC is carried out. Section 8 provides the conclusions.

## 2. Brief Overview of Delay Line TDCs

The time-to-digital converters (TDCs) are devices that convert an input time interval *T_In_* to a digital code word. Since this paper is focused on successive approximation TDCs that can be viewed as binary-scaled delay line TDCs, below the main characteristics of delay line TDCs are summarized.

**Figure 2:**
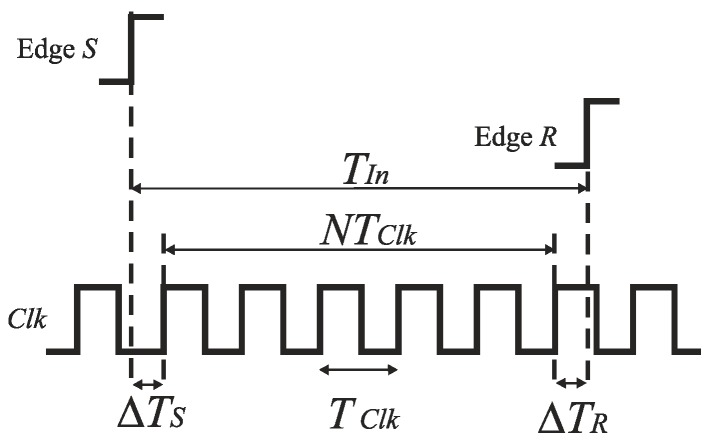
Time-to-digital conversion in counter-based TDC.

An early technique for a direct time digitization was based simply on counting a number of high frequency clock cycles *T_Clk_* during an input time interval *T_In_* defined by the edges (*S* and *R*) of a binary signal (Figure 2) [2,10]. The conversion time of the counter-based TDCs equals zero. The quantization noise introduced by the clock in such TDC is higher by 3 dB than in conventional ADCs if the start of the input time interval (edge *S* in Figure 2) is not synchronized with the reference clock [42]. The quantization step of the counter-based TDC is defined by the clock frequency (e.g., a time resolution 1 ns needs a clock frequency 1 GHz). The increase of the time resolution requires a higher frequency clock, and thus an increase of power consumption. On the other hand, the design of high-performance, high-frequency oscillators is limited by properties of submicron CMOS technologies [2].

**Figure 3:**
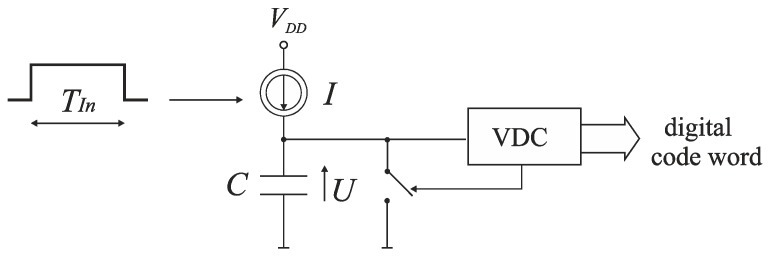
Diagram of TDC based on voltage-to-time translation.

The other early approach to the time-to-digital conversion consists in a translation of the input time interval *T_In_* to a corresponding voltage value, which is subsequently digitized by a classical voltage-to-digital converter (VDC) (Figure 3). First, a capacitor *C* is charged by a current source *I* during the time interval *T_In_*. Next, the voltage *U* on the capacitor is converted by the VDC to the digital word. The conversion accuracy depends on the linearity of time-to-voltage translation and the resolution of VDC [1,2]. The disadvantage of this technique is a relatively high-power consumption and a need to perform the voltage-to-digital conversion which increases a TDC conversion time and becomes more difficult in low voltage applications.

**Figure 4:**
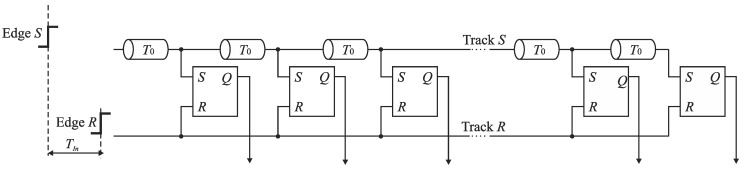
Diagram of time coding delay line TDC.

The need of fine time resolution in many applications has resulted in a development of TDC architectures based on the propagation delay of CMOS logic gates. One of generic TDC architectures that achieves a picosecond resolution is the *time coding delay line TDC* built of delay components and time comparators (e.g., RS latches, or D flip-flops) (Figure 4) [1,2,10,12]. The direct conversion process is based on successive delaying an event that represents a start of an input time interval (edge *S*) through a sequence of delay lines that introduce the same latency *T*_0_. In each step, the edge *S* delayed by *T*_0_ arrives to the input *S* of the RS latch acting as the time comparator. The output *Q* of each RS latch records the order in which the edges *S* and *R* arrive to its inputs. The conversion result is determined by the states of the outputs of RS latches and represented in the thermometer code. The digital equivalent of *T_In_* is equal to *kT*_0_ with a quantization error upper bounded by *T*_0_ where *k* is a number of high logic states at outputs of the RS latches. One of generic design solutions is that the delay line with a unit delay *T*_0_ is built of a pair of inverters. The quantization step *T*_0_ is thus limited by the feature size of CMOS technology.

**Figure 5:**
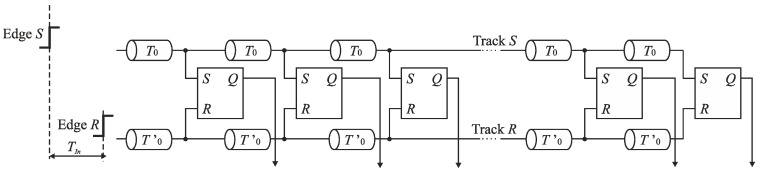
Diagram of Vernier delay line TDC.

The time resolution of the TDC with time coding delay line presented in Figure 4 can be improved by the use of the Vernier principle in fully integrated TDCs (Figure 5) [1,2,10,12,21]. In each conversion step, the edge *S* is delayed by Δ*T*_0_ in relation to the edge *R*, where Δ*T*_0_ = *T*_0_ − *T*’_0_ defines the converter resolution. The different values of delay units *T*_0_ and *T*’_0_ can be implemented by the design of two pairs of inverters with various *W*/*L* ratios for transistor sizes.

The *n*-bit TDC architectures presented in Figure 4 and Figure 5 have some significant drawbacks. The *n*-bit time-to-digital conversion is realized in a number of 2*^n^* steps and needs 2*^n^* time comparators. These numbers double with an increase of the resolution by one bit. The number of delay components required is 2*^n^* for the TDC with time coding delay line, and respectively 2∙2*^n^* for the TDC with Vernier delay line. The use of a large number of delay lines implies accumulation of jitter in further conversion steps of time-to-digital conversion. The digital output is obtained in the thermometer code which needs thermometer-to-binary conversion consuming a significant power and die area. These disadvantages can be alleviated by using the TDC based on successive approximation scheme (SA-TDC—Successive Approximation Time-to-Digital Conversion).

## 3. Schemes of Successive Approximation in Analog-To-Digital Conversion

**Figure 6:**
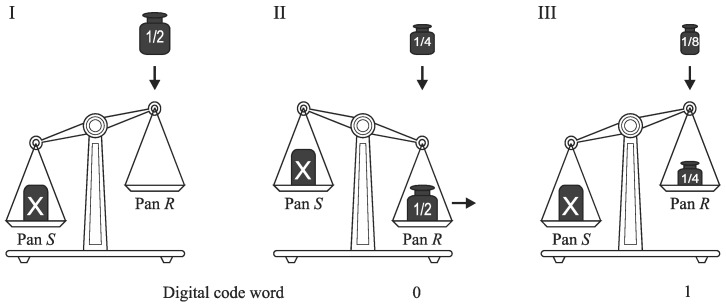
Illustration of oscillating successive approximation. (**I**),The mass *X* is placed in the pan *S*, and the weight is put on the pan *R*; (**II**), The accumulated mass on the pan *R* is larger than the mass *X*, the recent weight is removed and the subsequent binary-scaled weight is put on the pan *R*; (**III**), The accumulated mass on the pan *R* is smaller than the mass *X*, the recent weight is not removed and the subsequent binary-scaled weight is put on the pan *R*.

**Figure 7:**
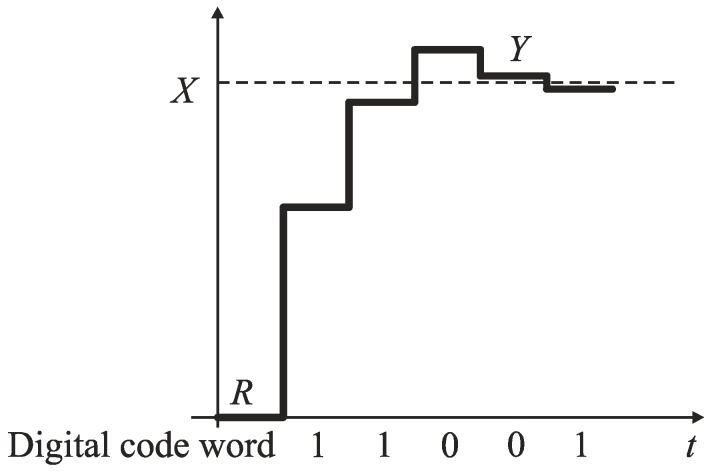
Oscillating successive approximation.

The successive approximation scheme belongs to fundamental and most successful methods of analog-to-digital conversion that has been implemented commercially for decades and is still used nowadays. Usually, the successive approximation is realized by *oscillating* or *monotone* algorithm [22]. Most ADCs for voltage input use the oscillating successive approximation (e.g., well-known ADC with charge redistribution [43]). Figure 6 shows an illustration how the oscillating successive approximation is realized using an analogy of weighting process of the unknown mass *X* by the use of a pan balance with a set of binary-scaled weights. Unknown mass *X* is placed in the pan *S*, and the weights are added always on the pan *R*. Before using a subsequent weight, it is necessary to check if the whole accumulated mass on the pan *R* is not larger than that on the pan *S*. If the total mass of the binary-scaled weights is larger than the input, the most recent weight is removed after each step. By the use of the oscillating scheme, the input *X* is successively approximated by its equivalent *Y* created in an oscillating way (Figure 7).

For some physical magnitudes however removing an already added weighting component is impossible or inconvenient. For example, the oscillating successive approximation cannot be applied to direct time-to-digital conversion although it may be performed indirectly by a prior translation of the input time interval to another magnitude (e.g., charge, voltage) which is further digitized [44] (time-to-digital conversion algorithm based on successive absolute difference operation between two time intervals is presented in [25,26]).

**Figure 8:**
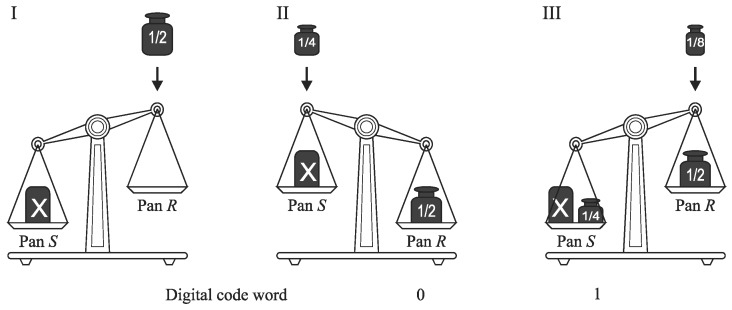
Illustration of monotone successive approximation. (**I**),The mass *X* is placed in the pan *S*, and the weight is put on the pan *R*; (**II**), The accumulated mass on the pan *R* is larger than the mass *X*, the subsequent binary-scaled weight is put on the pan *S*; (**III**), The accumulated mass on the pan *S* is larger than the accumulated mass on the pan *R*, the subsequent binary-scaled weight is put on the pan *R*.

The monotone successive approximation is a subtraction-free algorithm [22]. Its operation is illustrated also by the use of analogy of weighting process (Figure 8). In the first step, the weight corresponding to the half of the full scale is added on the pan *R* which is opposite to that where the unknown mass *X* has been put (*S*).

**Figure 9:**
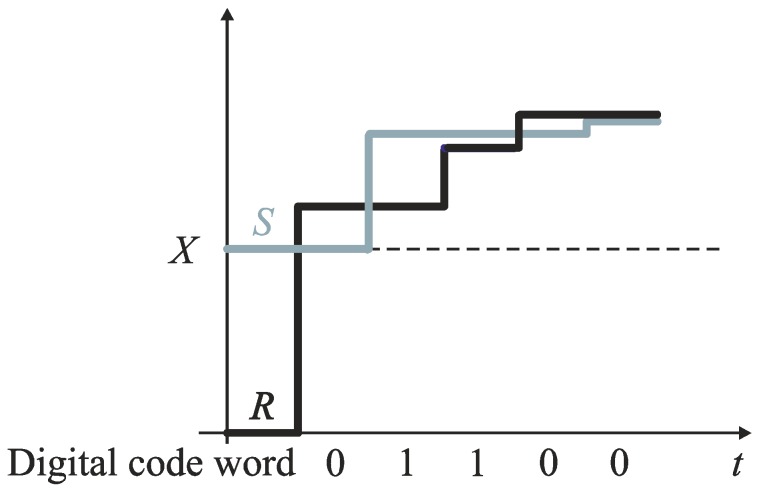
Monotone successive approximation.

In the next steps, each weight is added to this pan (*S* or *R*) that carries actually lighter total mass, so the total mass of each pan can only increase, or remain unchanged. The subtraction operation is thus eliminated. The mass *X* is determined with accuracy to a quantization step by the difference between the total mass of weights added on the pan *R* and respectively on the pan *S*. The output bits are evaluated successively after each conversion step. If *k*th weight was placed on the pan *R*, and the total mass at this pan is greater than the total mass of the pan *S*, then a corresponding bit *b_k_* is evaluated to ‘zero’. Otherwise, bit *b_k_* is set to ‘one’. For bits *b_k_* corresponding to weights collected on the pan *S*, the opposite annotation is used. In the monotone successive approximation, the values of both *S* and *R* increase monotonically (Figure 9), which is convenient for direct analog-to-digital conversion of physical magnitudes that are inherently increasing (e.g., time). However, the monotone successive approximation is also applied to analog-to-digital conversion in the voltage domain [45].

## 4. Time-to-Digital Conversion Based on Monotone Successive Approximation Scheme

Applying the monotone successive approximation scheme to a time-to-digital conversion led to the development of successive approximation time-to-digital converters (SA-TDCs) [27,28,29,30]. The principle of the SA-TDC is based on successive delaying the events defining a start and a stop of the input time interval *T_In_* similarly as for time coding delay line TDC (Figure 4). However, delaying the events is realized by the use of binary-scaled instead of uniform delays as is in time coding delay line TDCs. In each conversion step, the corresponding delay component is always introduced to this event which arrives earlier, so that both events at the end of the conversion coincide in time with a unit delay (*LSB*) resolution. The model of SA-TDC architecture introduced to the technical literature in [28,29,30] is of a feedforward type in contrast to the conventional SA-ADCs (Successive Approximation ADCs) that use architectures based usually on feedback [43].

**Figure 10:**
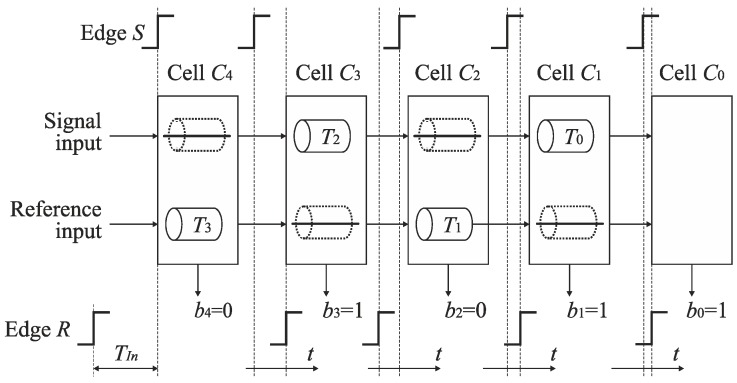
Conceptual model of feedforward type SA-TDC architecture for 5-bits.

The operation of the SA-TDC will be explained for *n* = 5 bits of resolution using a conceptual model shown in Figure 10. The SA-TDC consists of a chain of *n* = 5 cells. An input time interval *T_In_* is represented by a time distance between two events: a signal event *S* provided to a signal input, and a reference event *R* led to a reference input of the SA-TDC. Both events can be defined by sharp edges of a binary signal. The edges *S* and *R* are propagated through the converter in separate paths (track *S* and track *R*) that include delay lines *T*_3_, *T*_2_, *T*_1_, *T*_0_ located in the cells *C*_4_, *C*_3_, *C*_2_, *C*_1_. The presented SA-TDC is adapted to convert the bipolar input time intervals (when the order to events *S* and *R* is unknown a priori). If the rising edge of signal *S* precedes the rising edge of signal *R*, then the produced digital code word is positive (*MSB* = 1). Otherwise, it is negative (*MSB* = 0). The role of the delay lines is to introduce delays to the tracks *S* and *R*, respectively equal to *T*/4, *T*/8, *T*/16, *T*/32 where *T* is an input full scale of the SA-TDC. The output bit *b_i_*, where *i* = 0, 1, …, 4, is set to ‘one’ if the edge *S* enters the cell *C_i_* before the edge *R*. Otherwise, the bit *b_i_* is evaluated to ‘zero’. The cell *C*_0_ does not include any delay line because a state of the bit *b*_0_ (*LSB*) is determined by the order of edges *S* and *R* arriving to its inputs.

**Figure 11:**
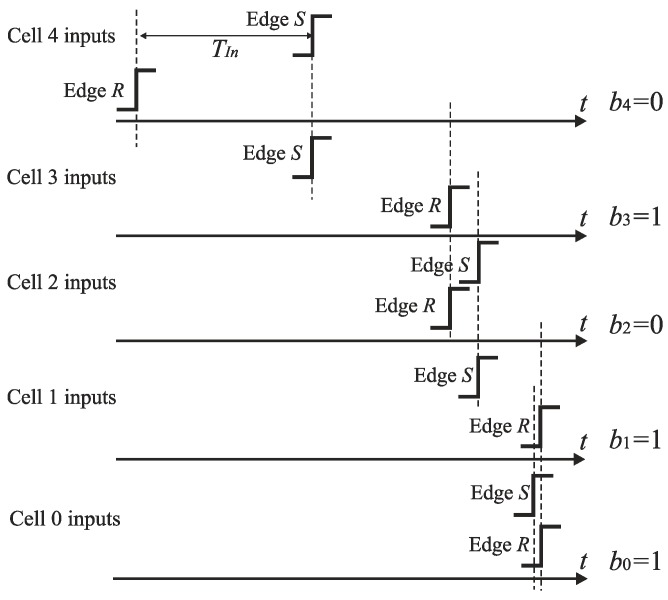
Time diagram of time-to-digital conversion for 5-bit SA-TDC.

Let us assume that a reference edge *R* arrives before a signal edge *S* to the inputs of the SA-TDC (Figure 11). Therefore, the bit *b*_4_ (*MSB*) is set to ‘zero’, and the edge *R* as an earlier event is directed to the delay line *T*_3_ with a latency equal to *T*/4. Assume that after delaying the edge *R* in the cell *C*_4_, the signal edge *S* precedes the reference edge *R* at the input of the cell *C*_3_. Then, the bit *b*_3_ is evaluated to ‘one’, and the edge *S* is provided to the delay line *T*_2_ having the latency *T*/8. Furthermore, if at the input of the cell *C*_2_, the reference edge *R* arrives before the signal edge *S*, the bit *b*_2_ is set to ‘zero’, and the edge *S* is provided to the delay line *T*_1_ with the latency *T*/16. If the reference edge *R* precedes again the signal edge *S* at the inputs of the cell *C*_1_, the edge *S* is directed to the delay line *T*_0_ with latency *T*/32. Finally, if the edge *S* arrives before the edge *R* to the cell *C*_0_, the bit *b*_0_ (*LSB*) is evaluated to ‘one’.

## 5. Basic SA-TDC Architecture

The concept of the successive approximation time-to-digital conversion has been initially implemented using the feedforward architecture [27,28,29,30].

### 5.1. SA-TDC Architecture for Bipolar Input

**Figure 12:**
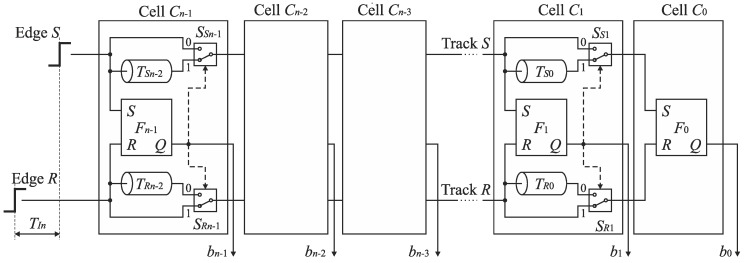
Basic *n*-bit feedforward SA-TDC architecture for bipolar input time interval *T_In_*.

The diagram of the basic feedforward SA-TDC architecture is presented in Figure 12 [22]. The *n*-bit SA-TDC consists of a cascade of *n* cells *C*_*n*−1_, …, *C*_0_, and each cell *C_k_* produces an output bit *b_k_* in the order from *MSB* to *LSB* where *k* = 0, …, *n*−1. The cells *C*_*n*−1_, …, *C*_1_ are equipped with a pair of delay lines (*T_Ri_*, *T_Si_*), a time comparator designed as an RS latch *F_i_*, and a pair of switches (*S_Ri_*, *S_Si_*) where *I* = 1, …, *n*−1. The cell *C*_0_ includes only the time comparator (RS latch) *F*_0_. The presented architecture refers to idealized conditions where the propagation time of signals by the multiplexers and RS latches is zero.

The edges *S* and *R* are propagated by the cells of SA-TDC in sequence from *C*_*n*−1_ to *C*_0_. The first role of cells *C*_*n*−1_, …, *C*_0_ is to recognize which rising edge (*S* or *R*) arrives to the inputs of cell *C_k_* earlier. The second role of cells *C*_*n*−1_, …, *C*_1_ is to delay the leading edge of signal (*S* or *R*) by a delay line contained in particular cells. The latency introduced by delay lines in cell *C*_*k*−1_ is always twice lower than in the preceding cell *C_k_*. The delay corresponding to the *LSB* is introduced by cell *C*_1_. The cell *C*_0_ does not include any delay lines. The aim of the last conversion step is to recognize which rising edge (*S* or *R*) reaches the input of cell *C*_0_ earlier and to define the *LSB*. As we discussed before, the SA-TDC in Figure 12 is a bipolar converter with full scale equal from −*T* to *T*. Thus, the cell *C*_*n*−1_ contains the delay lines (*T_Rn−2_*, *T_Sn−2_*) with propagation delay equal to 1/4 of full scale.

**Figure 13:**
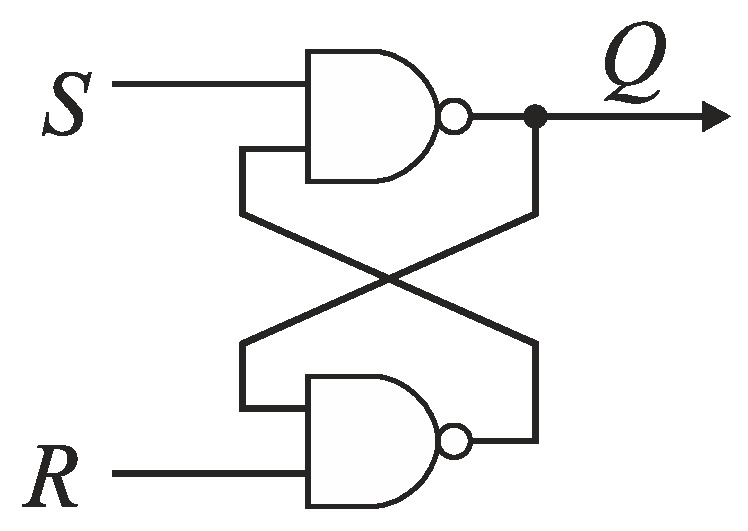
Time comparator *F_k_* based on RS latch.

The detection of this rising edge (*S* or *R*) that arrives to the input of cells *C*_*n*−1_, …, *C*_0_ as the first is carried out by the RS latch *F_k_* (Figure 13). If the states on RS latch inputs (*S* and *R*) are low, then an output *Q* of the latch *F_k_* is kept high. If a rising edge of the reference *R* precedes the edge of signal *S*, then *Q* becomes low. Otherwise, *Q* state is high. The output *Q* is used to control the pair of switches *S_Ri_* and *S_Si_*. The closure of one of switches allows to guide the leading edge of signal (*S* or *R*) to the delay line.

### 5.2. SA-TDC Architecture for Unipolar Input

**Figure 14:**
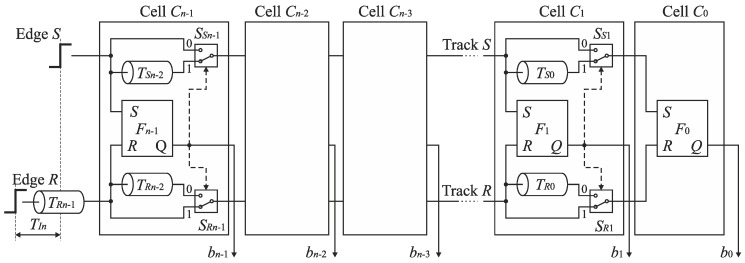
Basic *n*-bit feedforward SA-TDC architecture for unipolar input time interval *T_In_*.

The architecture of the SA-TDC presented in Figure 12 can be simply modified to convert the input time interval *T_In_* between the rising edges S and R if they appear in a priori known and predefined order. If the edge R always precedes the edge S at the inputs of the converter, then the first delay line *T_Rn−1_* with latency corresponding to 1/2 of conversion range is introduced in the track R. The SA-TDC shown in Figure 14 produces the unipolar digital code word corresponding to absolute value of the input time interval *T_In_*.

### 5.3. Related Works

The principle of SA-TDC based on monotone successive approximation has been invented by Edel and Maevsky [27] and developed further in [28,29,30]. The architectures of SA-TDCs are mostly of a feedforward type ([22,23,24,28,29,30,31,32]) so they include the number of time comparators equal to the number of conversion steps. The binary-scaled delay components are designed usually as chains of inverter pairs [30]. The application of feedforward SA-TDC in a low-density parity check (LDPC) decoder implemented in 65 nm CMOS technology is reported in [31].

On the other hand, the SA-TDC with feedback-based architecture has been introduced in [33] and further developed in [34,35]. The adoption of the feedback-based rather than feedforward architecture for *n*-bit SA-TDCs is motivated by possible reduction of the number of time comparators from *n* to one. The feedback-based SA-TDC architecture contains two loops for the events (i.e., reference and signal) being recycled and successively delayed by binary-scaled latencies.

An important issue of the feedback-based SA-TDC architecture is a problem to guarantee equal logic propagation delays introduced by the extra delay lines *T_m_* and multiplexers in both feedback loops. To cope with the problem of possible different delays for the events, a long offset time to both loops has been used in [33]. Unfortunately, an extra offset delay in each conversion step of the feedback-based SA-TDC increases the conversion time, which becomes then much longer than in case of the feedforward SA-TDCs. To achieve the conversion time of the feedback-based SA-TDC equal to that of feedforward SA-TDC, a concept of dynamic equalization of logic propagation delays in both loops of the feedback-based SA-TDC architecture has been proposed in [36]. The architecture of the feedback-based SA-TDC with dynamic delay equalization is studied through extensive simulation analysis in [37]. The implementation of feedback-based SA-TDC with 1.2 ps quantization step and 328 μs dynamic range in 0.35 μm CMOS technology is presented in [33].

Some propositions of new successive approximation algorithms in the time domain appeared recently. In order to simplify the architecture of SA-TDC cells, Decision-Select Successive Approximation (DSSA) algorithm as a modification of the monotone successive approximation has been proposed and implemented in 65 nm CMOS technology [32]. In DSSA, only one signal (input signal) is guided to the delay lines based on time comparators outputs. The other (reference signal) is delayed in each cell regardless of the time comparator decisions. A new successive approximation algorithm in time domain, called Successive Approximation with Continuous Disassembly (SACD) has been reported in [25,26]. In SACD algorithm, the input to each conversion step is the absolute difference by using XOR operation between the input and the binary-scaled weight that corresponds to the previous step. Furthermore, the output correction process is needed in which the value of each bit is used to correct the value of the next one by a simple digital logic. This study is an extended version of previous papers that presented contributions on the design and optimization of feedforward SA-TDCs [22,23,24].

## 6. Optimization of Basic SA-TDC Architecture

The basic architecture of *n*-bit SA-TDC shown in Figure 12 can be optimized in terms of a number of logic gates.

### 6.1. SA-TDC with Single Set of Delay Lines

**Figure 15:**
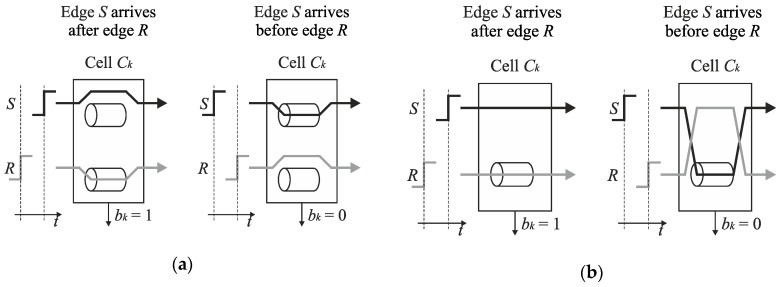
Illustration of the use of delay lines in feedforward SA-TDC architecture (**a**), and a concept of SA-TDC architecture with a single delay line in each cell (**b**).

An analysis of the operation of the conversion algorithm shows that the number of delay lines in the basic SA-TDC architectures (Figure 12 and Figure 14) is redundant because only one delay line, located in track *S*, or in track *R*, is used in each conversion step (Figure 15a).

**Figure 16:**
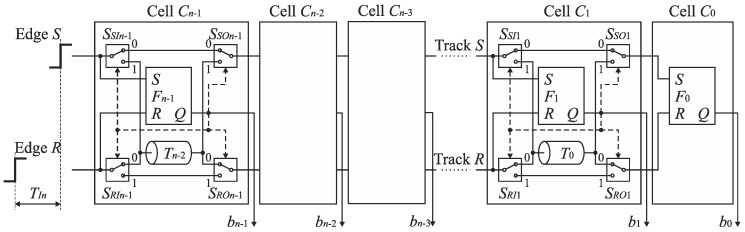
Architecture of *n*-bit feedforward SA-TDC with single set of delay lines.

Therefore, the cells *C*_*n*−1_, …, *C*_1_ can be equipped only with a single delay line included in track S or R at the price of a little extension of control logic [23]. Figure 15b shows the SA-TDC with the delay lines located in the track R. If the edge *S* precedes the edge *R* at the input of the cell *C_k_*, then it has to be guided to the delay line located in the track R. For this purpose, each cell *C*_*n*−1_, …, *C*_1_ must be equipped with an extra pair of switches *S*_*SIn*−1_, *S*_*RIn*−1_, …, *S_SI_*_1_, *S_RI_*_1_ (Figure 16) in order to guide a front edge (*S* or *R*) to a single delay line located in one of tracks (in track *R* in Figure 16). A role of the output pair of switches *S*_*SOn*−1_, *S*_*ROn*−1_, …, *S*_*SO*1_, *S*_*RO*1_ is to restore an original track for the propagated edges before they enter the inputs of the next cell.

### 6.2. SA-TDC with Single Set of Delay Lines and Output Decoding

**Figure 17:**
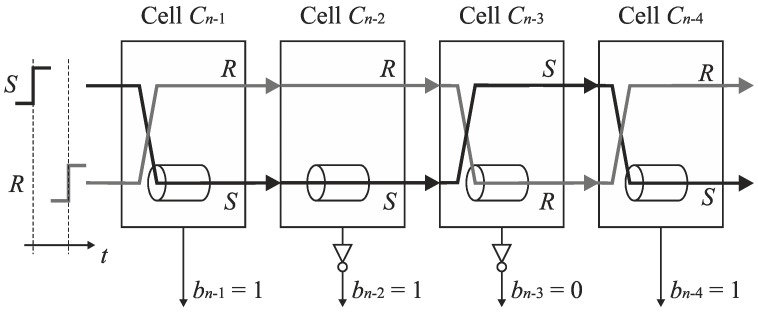
Propagation of signals *S* and *R* in *n*-bit feedforward SA-TDC with single set of delay lines without output switches.

Further analysis of delay line swap shows that the output pair of switches *S*_*SOn*−1_, *S*_*ROn*−1_, …, *S*_*SO*1_, *S*_*RO*1_ can be eliminated from the converter architecture presented in Figure 16 at the price of simple output decoding. Note that if the output pair of switches *S_SOk_*, *S_ROk_* is removed from the cell *C_k_*, then the edges *S* and *R* come to the inputs of the next cell *C*_*k*−1_ in the opposite tracks in case if they have altered the tracks in the cell *C_k_*. Although the RS latch *F*_*k*–1_ in the cell *C*_*k*−1_ recognizes which edge arrives earlier even if the edges arrive to the inputs of cell in opposite tracks, the output bit *b*_*k*−1_ is set then to an inverted state. The corresponding principle is presented in Figure 17. Assume that the edge *S* precedes the edge *R* at the input of the cell *C*_*n*−1_. Then, the edge *S* is directed to the delay line located in the track *R*, and the bit *b*_*n*-1_ corresponding to MSB is evaluated to ‘one’. If the pair of output switches is removed, then the edges *S* and *R* are swapped, that is, they occur respectively in the track R and S at the input of cell *C*_*n*−2_. Assume that the edge *S* (propagated in track *R*) follows the edge *R* (propagated in track *S*) at the input of the cell *C*_*n*−2_. Subsequently, the output bit *b*_*n*−2_ is set to ‘zero’ instead of to ‘one’ because the edges (*S* and *R*) are propagated in the opposite tracks at the input of the cell *C*_*n*−2_. Hence, in order to recover a correct state, the bit *b*_*n*−2_ has to be inverted. Next, let us assume that the edge R precedes the edge *S* at the input of cell *C*_*n*−3_. Then, the edge *R* is directed to the delay line. The inversion of the state of the bit *b*_*n*−3_ is also needed because the edges (*S* and *R*) come to the inputs of the cell *C*_*n*−3_ in altered tracks. Since the edge *R* has been guided to the delay line, an original track for the propagated edges are restored before they enter the inputs of cell *C*_*n*−4_. Hence, the inversion is not needed for the bit *b*_*n*−4_.

**Figure 18:**
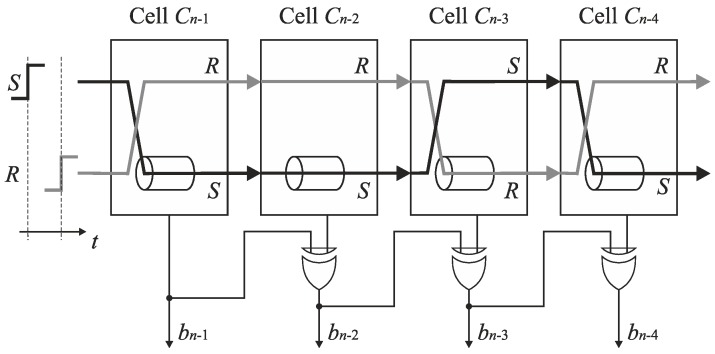
SA-TDC with single set of delay lines and without output pair of switches.

To sum up, the correct evaluation of SA-TDC output bits is possible even if the signals *S* and *R* are not propagated permanently through the tracks *S* and *R*. When the edges arrive to the inputs of the cells *C*_*n*−2_, …, *C*_0_ in opposite tracks, then the states of the bits *b*_*n*−2_, …, *b*_0_ have to be inverted. This principle does not apply to the bit *b*_*n*−1_ because the edges (*S* and *R*) by assumption reach the input of the cell *C*_*n*−1_ in the predefined order (for unipolar input). The signals occur in the opposite tracks when the number of track swap is odd. The inversion is required for these output bits that are between odd and even occurrences of the bits whose state is set to ‘one’. The detection of an odd number of track swap and decoding of a digital output code word may be performed by a decoder presented in Figure 18. Getting correct states of output bits *b*_*n*−2_, …, *b*_0_ needs to equip the cells *C*_*n*−2_, …, *C*_0_ with a simple decoder based on XOR gates.

**Figure 19:**
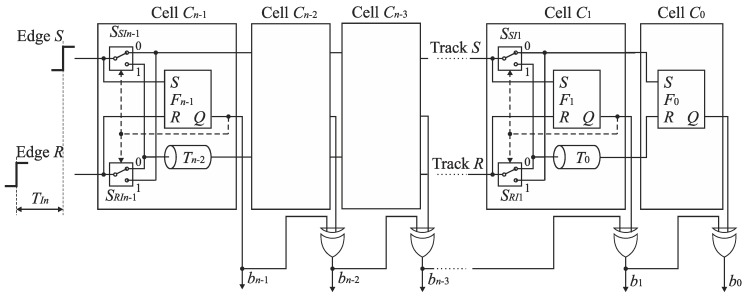
Architecture of *n*-bit feedforward SA-TDC with single set of delay lines and output decoding.

The optimized architecture of n-bit SA-TDC with single set of delay lines and output decoding is shown in Figure 19 [23]. Elimination of output switches is advantageous due to reduction of circuit complexity. Furthermore, since the XOR gates are located outside tracks *S* and *R*, they have no impact on mismatch of propagation delays in tracks *S* and *R*.

### 6.3. Compensation of Logic Propagation Delays

The idealized SA-TDC architectures shown in Figure 12, Figure 14, Figure 16 and Figure 19 have been developed based on the assumption that digital logic components (i.e., switches, RS latches) operate with infinite speed. In practice, the time needed to produce an output for digital gates is non-zero, and may be relatively long. In particular, it refers to the time comparators when the edges *S* and *R* to its inputs quasi-simultaneously (see Section 7.2). In order to guarantee a reliable converter operation, the cells have to be equipped with extra delay lines aimed to compensate logic propagation delays.

**Figure 20:**
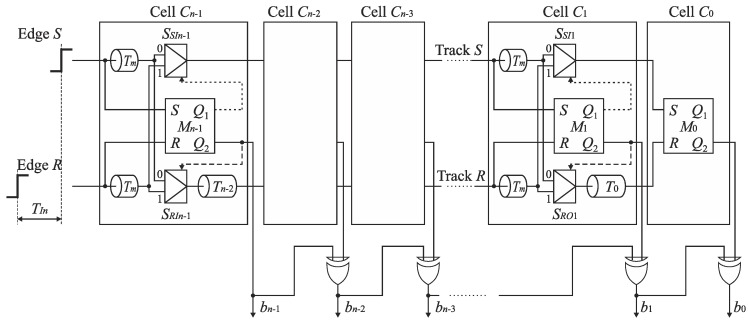
Architecture of *n*-bit feedforward SA-TDC with single set of delay lines and compensation of logic propagation delays.

**Figure 21:**
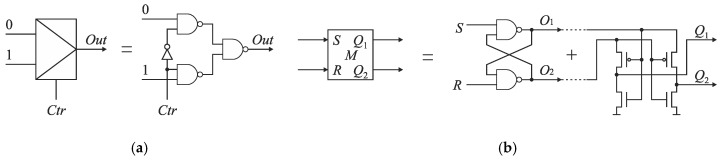
Diagram of: (**a**) two-to-one multiplexer; (**b**) MUTEX block.

The SA-TDC architecture with compensation of logic propagation delays is presented in Figure 20. The switches have been designed as two-to-one multiplexers (Figure 21a), the time comparators are MUTEX blocks (Figure 21b), and the delay lines are built of chains of pairs of inverters. The objective of the extra delay lines *T_m_* is to ensure that the propagated edges arrive to the multiplexer inputs when the MUTEX block has produced the output, and when this signal has already come to the multiplexer control input implying to switch the relevant channel of the multiplexer (Figure 20). The value of *T_m_* should be long enough to compensate the response time of MUTEX block and switching time of multiplexers. However, too long *T_m_* value increases the SA-TDC conversion time, requires the use of redundant inverters, and introduces higher conversion errors due to *T_m_* delay mismatch in fabrication process. Therefore, the value of *T_m_* should be a tradeoff between converter performance on one hand, as well as the conversion time and chip die area on the other.

### 6.4. Evaluation of SA-TDC Circuit Complexity by Proposed Design Optimization

The reduction of SA-TDC complexity between the basic feedforward architecture with two sets of delay lines, and the feedforward architecture with single set of delay lines and output decoding, can be evaluated by comparison of the number of transistors used to build both version of the converter. We assume that a multiplexer is built of $T_{Mux}=28$ transistors, a time comparator (MUTEX) respectively of $T_{TCOmp}=12$, while XOR gate of $T_{XOR}=12$ transistors. The number of transistors in one set of delay lines is 4 + 8 + … + 2^*n* + 1^, and in logic delay compensation, respectively, $2\left(n-1\right)T_{mn}$ where $T_{mn}$ is the number of transistors in a single delay line *T_m_*. Therefore, the number of the transistors in the *n*-bit SA-TDC with two sets of delay lines is:

$$N_{1}=2\cdot \left(n-1\right)\cdot T_{Mux}+n\cdot T_{TComp}+2\left(n-1\right)T_{mn}+\overset{n}{\underset{k=2}{\sum }}2^{k+1}$$

and for the *n*-bit SA-TDC with single set of delay lines and output decoding, respectively:

$$N_{2}=2\cdot \left(n-1\right)\cdot T_{Mux}+n\cdot T_{TComp}+\left(n-1\right)\cdot T_{XOR}+2\left(n-1\right)T_{mn}+\overset{n}{\underset{k=2}{\sum }}2^{k}$$

**Figure 22:**
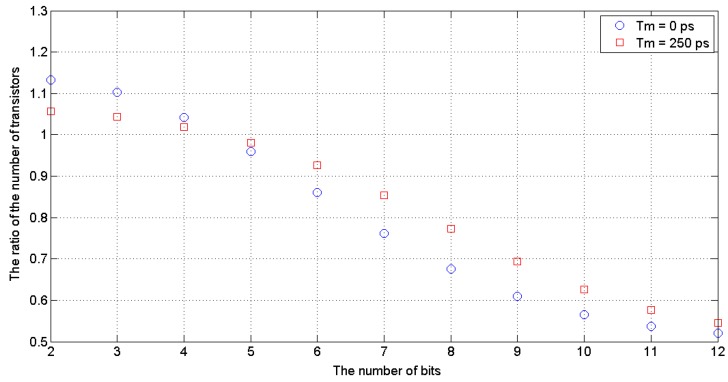
Reduction of SA-TDC complexity obtained by design optimization vs. number of bits.

Table 1 and Figure 22 show the relationship of the ratio $N_{1}/N_{2}$ versus the SA-TDC resolution (*n*) for *T_m_* = 0 and *T_m_* = 250 ps. For n>4, the number of transistors used to build the SA-TDC with single set of delay lines and output decoding is smaller than for the basic version with two sets of delay lines. For n=8, the reduction of complexity is around 30% for *T_m_* = 0 and around 20% for *T_m_* = 250 ps, while for n=12, respectively, is almost 50%.

## 7. Implementation of SA-TDC in 180 nm CMOS Technology

The SA-TDC with single set of delay lines and output decoding (Figure 19) has been implemented for 8 bit of resolution in 180 nm CMOS technology. The design process is reported in details below.

### 7.1. Delay Lines

**Figure 23:**
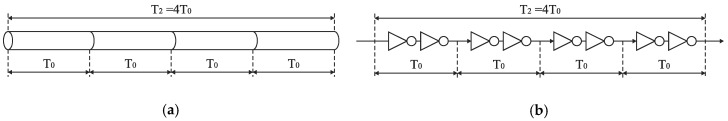
(**a**) Concept of delay lines design; (**b**) delay line *T*_2_ as a cascade of 4 inverters pairs.

The delay lines *T*_6_, …, *T*_0_ in 8-bit SA-TDC has been designed as a cascade of inverter pairs. The delay line *T*_0_ in the cell *C*_1_ is built of a single pair of inverters. In the other cells, the delay lines are doubled with the increase of the cell index (Figure 23).

**Figure 24:**
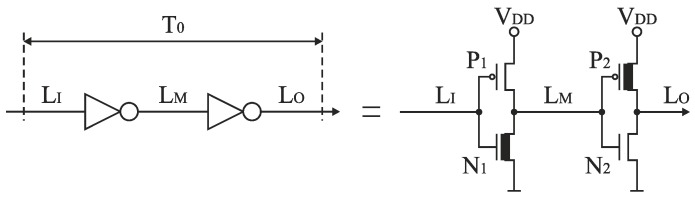
Asymmetric design of pair of inverters.

Alternatively, the delay lines could be implemented using differential delay components. Such implementation is less sensitive to power supply fluctuations, and allow to obtain better resolution compared to the delay lines based on the inverters. On the other hand, the differential delay cells are more sensitive to mismatch in fabrication process, consume more power, and require more die area. Since the primary design objective of the proposed approach is to reduce the SA-TDC die area, we have decided to use the delay lines based on the inverters. The propagation time of a single pair of inverters defines a quantization step *T*_0_ as a size of LSB of the SA-TDC. In [29,30], the propagation time of an inverter designed in 180 nm CMOS technology is around 50 ps, which gives *T*_0_ equal to 100 ps. In order to decrease a quantization step of the SA-TDC, the dimensions of transistors in the pair of inverters were shaped accordingly to transmit quickly only an active signal edge. As a result, the inverters in the pair have been designed asymmetrically and adapted to deal with the active rising edges (Figure 24).

**Figure 25:**
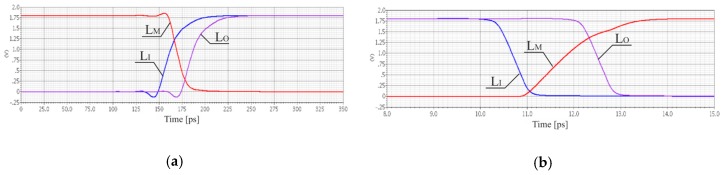
Simulation results for 180 nm CMOS process: signals at input (L_I_), in the middle (L_M_), and at output (L_O_) of asymmetric pair of inverters for rising active edge (**a**); signals at input (L_I_), in the middle (L_M_), and at output (L_O_) of asymmetric pair of inverters for falling inactive edge (**b**).

The first inverter is aimed to transmit quickly a rising edge of a signal, while the second inverter is adapted to introduce the minimum propagation latency for falling edge of the signal. The simulation experiment shows that the proposed solution allows to reduce the propagation time of the rising edge by a single pair of asymmetric inverters to 24.4 ps for the 180 nm CMOS technology. The value of *T*_0_ has been rounded to 25 ps by suitable W/L ratios of transistors (Table 2) and defines the quantization step of implemented 8-bit SA-TDC. The reduction of propagation time for rising active edges (Figure 25a) is obtained at the price of the increase of propagation time for falling inactive edges (Figure 25b). However, the latter does not imply any restrictions for converter operation except the necessity to increase the dead time between subsequent cycles of time-to-digital conversion. The similar method of the reduction of the active edge propagation time by the pair of inverters has been applied in [31].

### 7.2. Time Comparator

**Figure 26:**
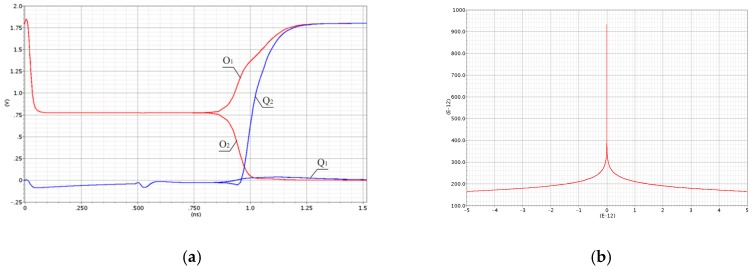
Metastable states at output *O*_1_ and *O*_2_ of RS latch (**a**). Response time of MUTEX vs. time interval at MUTEX input (**b**).

The MUTEX block acts as a time comparator and realizes mutual exclusion operation. The MUTEX consists of an RS latch and a metastability filter (Figure 21b). If the states of both inputs *S* and *R* are low, then the outputs *Q*_1_ and *Q*_2_ are driven to ‘zero’. If the edge *R* precedes the edge *S*, then *Q*_1_ and *Q*_2_ are respectively set to ‘zero’ and ‘one’. Otherwise, the *Q*_1_ and *Q*_2_ are respectively ‘one’ and ‘zero’. The role of metastability filter is to prevent the MUTEX outputs *Q*_1_ and *Q*_2_ from metastable states at the outputs *O*_1_ and *O*_2_ of the RS latch. The metastable states in the RS latch occur when the both edges (*S* and *R*) come to the inputs of the MUTEX block almost at the same time [46]. Then, the RS latch requires a long time to decide which edge arrived first, and is reflected by the metastable state on *O*_1_ and *O*_2_ (Figure 26a). The response time of the MUTEX implemented in 180 nm CMOS technology versus the input time interval is illustrated in Figure 26b based on the simulation experiment in Cadence. As seen in Figure 26b, the response time of the MUTEX for quasi-simultaneous edges of signals S and R can be very long.

### 7.3. Preliminary Tests of SA-TDC with T_m_ = 250 ps

**Figure 27:**
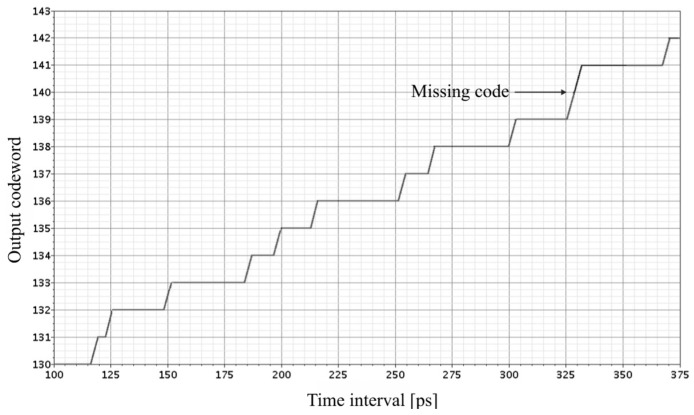
Transfer characteristics of 8-bit SA-TDC with *T*_0_ = 25 ps and *T_m_* = 250 ps.

In order to examine preliminarily the 8-bit SA-TDC performance with the quantization step *T*_0_ = 25 ps and the full scale ±*T* = 3.175 ns in 180 nm CMOS technology, the first simulation experiment has been run for *T_m_* = 250 ps. The simulation results for a fragment of a transfer characteristics of the 8-bit SA-TDC is presented in Figure 27 show that the transfer function of SA-TDC is incorrect because it contains a significant differential nonlinearity (DNL) and a missing digital code word around the value of 140.

To identify the reasons of losing the assumed converter resolution, the analysis of sources of conversion errors has been carried out. This analysis shows that apart from the mismatch of the binary-scaled delay lines, the primary source of conversion errors are non-zero propagation delays of digital logic components (i.e., MUTEX blocks and multiplexers) incorporated in the propagation tracks of the input edges. As mentioned, the MUTEX blocks suffer from metastability and may introduce a gross error if the response is produced after a very long time, which occur for quasi-simultaneous arrival of the edges (see Section 7.2) to any cell.

The other source of errors introduced by digital logic are multiplexers located in both tracks *S* and *R*. Ideally, the propagation delays of the multiplexers in both tracks should be the same. However, the propagation delays of the multiplexers differ because in each cell *C*_*n*−1_, …, *C*_1_ only the multiplexer (*S_Si_* or *S_Ri_*) located in the track of the earlier edge is switched. The other multiplexer located in the track of the later edge is not switched and keeps the connection already established.

**Figure 28:**
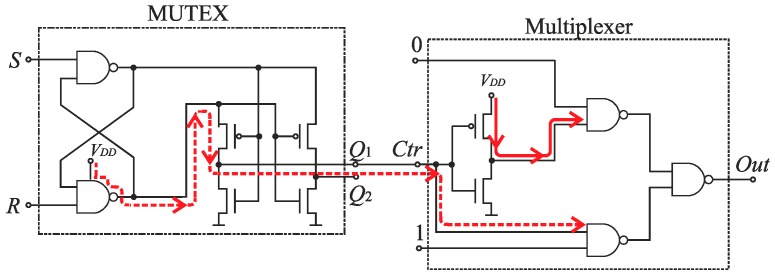
Asymmetrical control of channels (0 and 1) of multiplexer.

Furthermore, the next reason of various propagation delays introduced by multiplexers stems directly from design of the multiplexer (Figure 21a), which implies an asymmetrical control of both channels (0 and 1) (Figure 28). The input of a NAND gate in the channel 1 is driven from the MUTEX output *Q*_1_. On the other hand, the input of the NAND gate in the channel 0 is driven from the inverter output which is fed directly from the voltage source *V_DD_*. Therefore, the control signal in the channel 0 is stronger than in the channel 1, which implies longer time needed for multiplexer switching in the latter, and needs to use longer *T_m_* delays.

**Figure 29:**
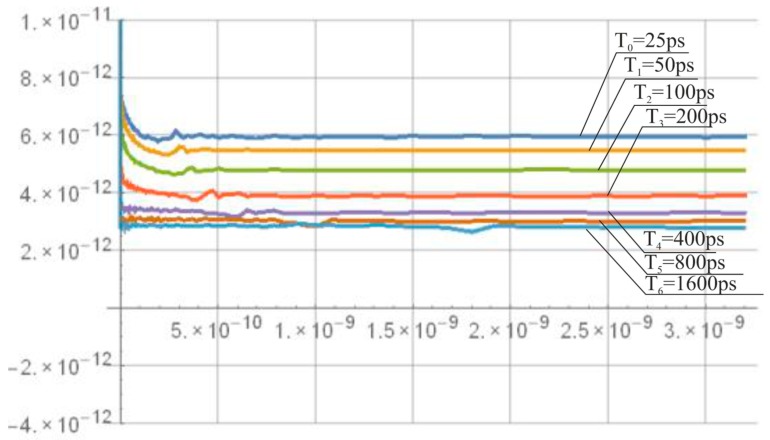
Differences of propagation delays of multiplexers *S_Si_* and *S_Ri_* vs. input time interval *T_In_* for 8-bit SA-TDC with *T_m_* = 250 ps evaluated for cells including various binary delays.

In order to estimate the errors introduced by multiplexers, the differences in propagation delays of the multiplexers *S_Si_* and *S_Ri_* versus the input time interval (*T_In_*) varying from 0 to *T* for all the cells separately have been evaluated by simulations. The plot of this relationship for particular cells of SA-TDC with *T_m_* = 250 ps is presented in Figure 29. For the same propagation times of multiplexers, the calculated difference in each cell should be zero. Instead, the simulation results show that the differences in propagation delays of the multiplexers vary from 3 ps for cells including long binary delays (*T*_0_ = 400 ÷ 1600 ps) to 6 ps for the cell *C*_1_ with 25 ps delay. Additionally, the differences of multiplexers propagation delays are higher for short *T_In_* than for long *T_In_*.

**Figure 30:**
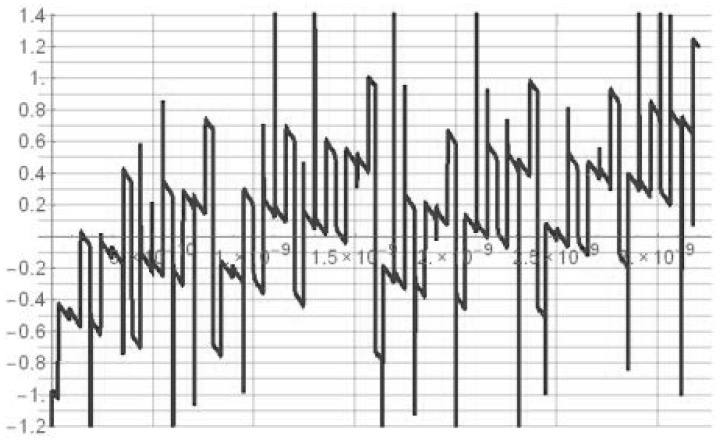
Total delay error normalized to *LSB* (*T*_0_) introduced by multiplexers *S_Si_* and *S_Ri_* vs. input time interval *T_In_* for 8-bit SA-TDC with *T_m_* = 250 ps.

The conversion errors resulted from different propagation delays of the multiplexers cumulate during further steps of conversion process and can exceed the *LSB* for some input values. The total delay error normalized to *LSB* (*T*_0_) which is introduced by the multiplexers *S_Si_* and *S_Ri_*, shown in Figure 30, is greater than *LSB* for some values of *T_In_*. This is a reason why the transfer characteristics of the SA-TDC includes missing code words (Figure 27). Thus, extra delay *T_m_* = 250 ps turned out too small to compensate a time needed by the multiplexers to switch the relevant track for propagated edges.

**Figure 31:**
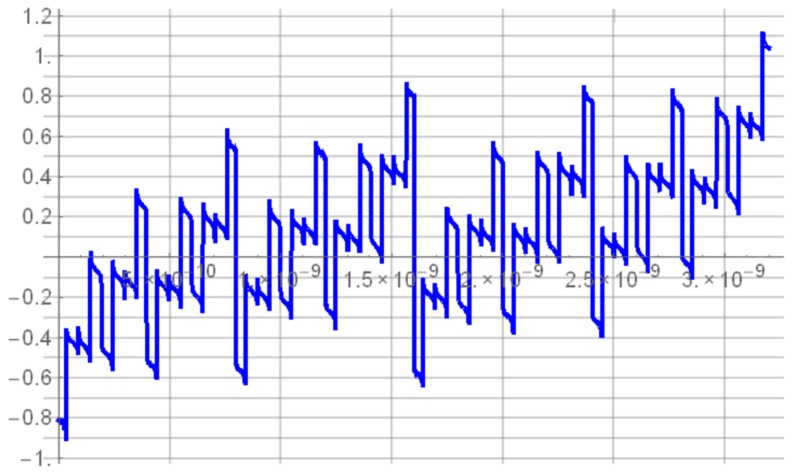
Total delay error normalized to *LSB* (*T*_0_) introduced by multiplexers *S_Si_* and *S_Ri_* vs. input time interval *T_In_* for 8-bit SA-TDC with *T_m_* equal to 350 ps.

**Figure 32:**
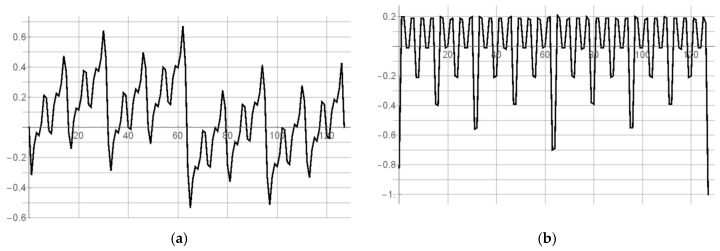
(**a**) INL normalized to *LSB* (**b**) DNL normalized to *LSB* for code words at 8-bit SA-TDC and *T_m_* = 350 ps.

To give additional time for switching of the multiplexers and reduce the conversion errors, the converter performance for *T_m_* > 250 ps was examined. The conclusion from the simulation tests is that the increase of *T_m_* to 350 ps allows to keep the total delay error introduced by multiplexers (Figure 31), as well as INL (Figure 32a) and DNL (Figure 32b) errors of the SA-TDC below *LSB* for the whole conversion range.

### 7.4. Reducing T_m_ Delay by Symmetrizing Multiplexer Design

Although the use of extra delay lines compensating digital logic latency allows to achieve the assumed 8-bit resolution of the SA-TDC, the additional delay that has to be used (*T_m_* = 350 ps) is relatively long (an order of magnitude longer than the quantization step *T*_0_ = 25 ps), which increases chip die area and conversion time. The reduction of *T_m_* delay is possible provided that the total conversion error can be further reduced. We decided to make the effort towards decreasing inequality of delays contributed by the multiplexers. This issue has been addressed by attempt to symmetrize the classical multiplexer design in order to equalize their propagation time.

**Figure 33:**
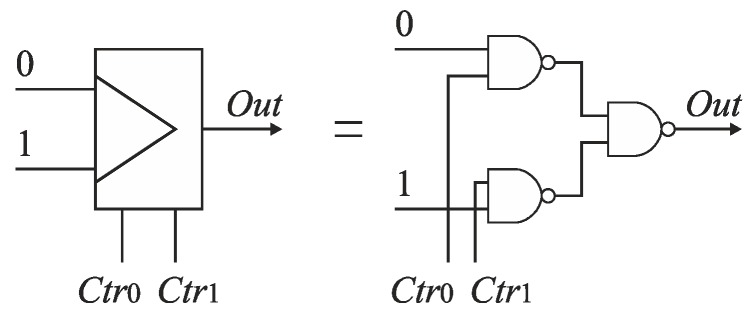
Diagram of symmetrized multiplexer design.

**Figure 34:**
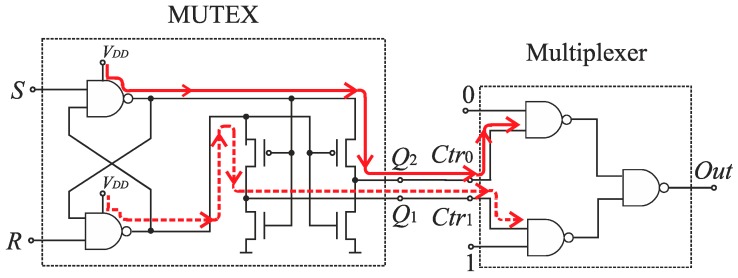
Symmetrical control of both channels of symmetrized multiplexer.

**Figure 35:**
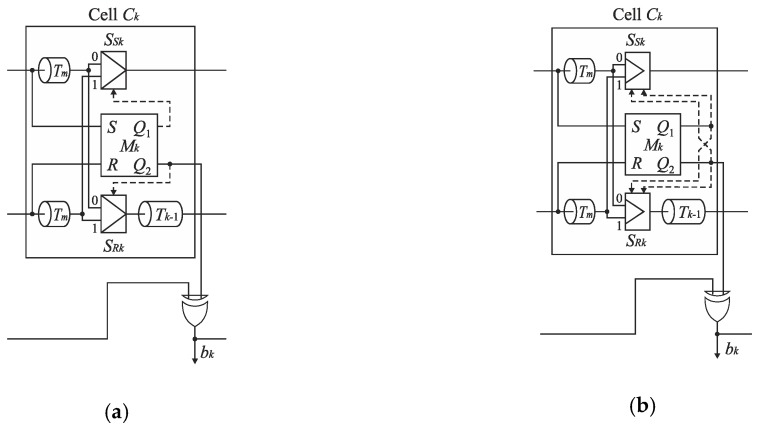
Cell of SA-TDC with classic (**a**) symmetrized (**b**) multiplexers design.

The classical multiplexer includes an inverter that introduces some design asymmetry (Figure 21a). To reduce the time of stabilization of the states on multiplexer outputs, the inverter has been removed from the multiplexer topology (Figure 33). Both channels (0 and 1) of the multiplexer are controlled directly from the MUTEX outputs, which are set to the opposite states. Therefore, the control of the channels is fully symmetrical (Figure 34). The use of symmetrical control of multiplexers in the SA-TDC requires to alter the connections between the MUTEX outputs and the multiplexers control inputs (Figure 35).

**Figure 36:**
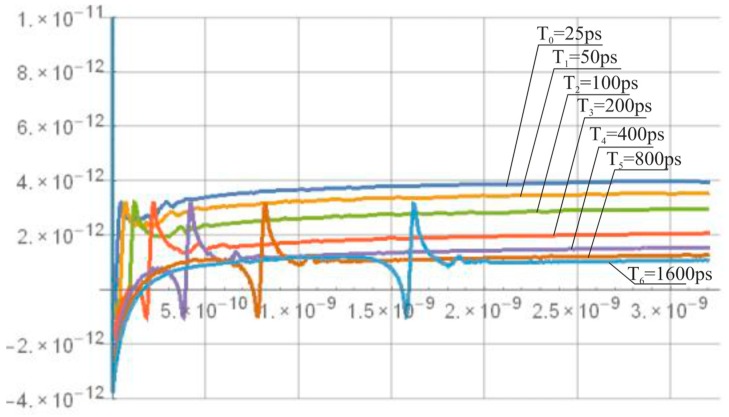
Differences of propagation delays of symmetrized multiplexers *S_Si_* and *S_Ri_* vs. input time interval *T_In_* with *T_m_* = 250 ps.

**Figure 37:**
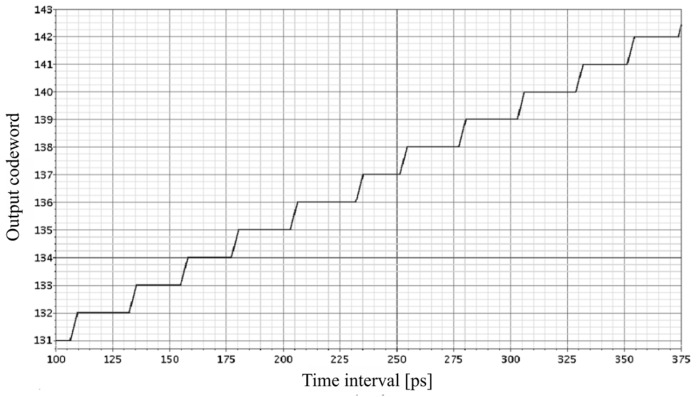
Transfer characteristics of 8-bit SA-TDC with symmetrized multiplexers for *T*_0_ = 25 ps and *T_m_* = 250 ps.

**Figure 38:**
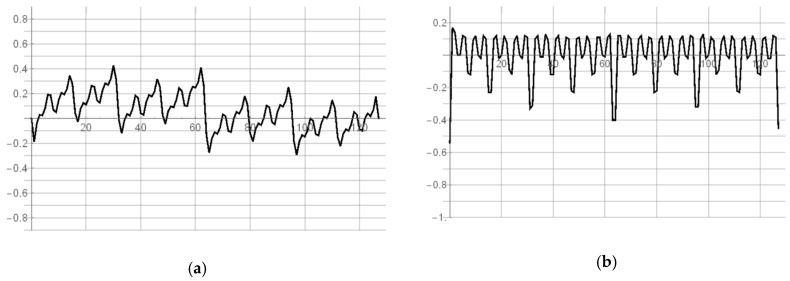
(**a**) INL normalized to *LSB* (**b**) DNL normalized to *LSB* for code words at 8-bit SA-TDC with symmetrized multiplexers and *T_m_* = 250 ps.

The impact of the use the symmetrized multiplexers on the 8-bit SA-TDC with *T_m_* = 250 ps was assessed based on analysis of the simulation results for the differences in propagation delays contributed by multiplexers *S_Si_* and *S_Ri_* with symmetrical control (Figure 36). In comparison to Figure 29, the inequalities of the propagation delays introduced by symmetrized multiplexers in the particular SA-TDC cells are lower than for the classical multiplexers used in the same conditions. With the symmetrized multiplexers, the transfer characteristics of the 8-bit SA-TDC for *T_m_* = 250 ps does not include missing code words (Figure 37). The results of the analysis of INL and DNL for the code words corresponding to the half of full scale are respectively presented in Figure 38a,b. As follows from these plots, the INL and DNL errors are below 1/2 *LSB*. The parameters of the SA-TDC design with symmetrical control of multiplexers are summarized in Table 3. Furthermore, the comparison of the design presented in this paper is in Table 4 referred to previous works on SA-TDCs.

### 7.5. Analysis of Device Mismatch and Time Jitter

The performance of SA-TDCs is limited by device mismatch in fabrication process and noise (time jitter). The nature of both phenomena, inherent to the physics of the transistor, is statistical. The time jitter is time-variant while mismatch is time-invariant because essentially the timing deviation of each unit delay from its nominal value does not change during circuit operation. The propagation time uncertainty through the *i*th unit element (pair of inverters), $T_{unit,i,0}$, due to devices mismatch is [37]:

$$T_{unit,i,0}=T_{0}+\Delta t_{mism,i,0}$$

where $T_{0}$ is the nominal unit delay, and $\Delta t_{mism,i,0}$ is the deviation from the nominal propagation time of the *i*th unit element implied by process mismatch. The deviation $\Delta t_{mism,i,0}$ can be modelled by the Gaussian random variable with zero mean and standard deviation $\sigma_{mism}$. Since the device mismatch of unit delay elements can be considered uncorrelated for each unit element, an edge propagating through a delay line built of a casade of *m* unit delays will experience a delay with mean equal to $\sum_{i=1}^{m}T_{unit,i,0}$ and standard deviation $\sqrt{m}\sigma_{mism}$.

The propagation time of an event through the *i*th unit element due to time jitter is [37]:

$$T_{unit,i,k}=T_{0}+\Delta t_{jitter,i,k}$$

where $\Delta t_{jitter,i,k}$ is the deviation from the nominal propagation time due to time jitter. The deviation $\Delta t_{jitter,i,k}$ can be modelled by the Gaussian random variable with zero mean and standard deviation $\sigma_{jitter}$, while *k* is the index of the edge propagating through such delay element. Since noise is a time-variant phenomenon, the timing deviation $\Delta t_{jitter,i,k}$ varies for each edge propagated through the *i*th delay element. As the noise of different unit delay elements can be considered uncorrelated and having the same standard deviation $\sigma_{jitter}$, an edge propagating through a delay line built of a casade of *m* unit delays (e.g., the first *m* elements) will experience a delay with mean equal to $\sum_{i=1}^{m}T_{unit,i,0}$ and standard deviation $\sqrt{m}\sigma_{jitter}$.

In general, any deviation from the nominal propagation time of delay elements is particularly harmful since it accumulates along the propagation of the event through the delay lines. In order not to excessively impair the effective resolution of the SA-TDC, the total accumulated time error must be smaller than the nominal unit delay $T_{0}$ (*LSB*).

Let us neglect the impact of *T_m_* on *n*-bit SA-TDC delay mismatch and time jitter. Assume also for the sake of simplicity that the input time interval equals approximately the half of the input full-scale ($T_{In}≅T/2$) with the edge *S* preceding the edge *R*. Then, the event *S* is propagated by *n*-1 delays components with the number of unit delay elements (pairs of inverters) equal to $2^{\left(n-1\right)}-1$, which corresponds to the total propagation time $\sum_{i=1}^{2^{\left(n-1\right)}-1}T_{unit,i,0}$. The standard deviation of the total propagation time error of the *n*-bit SA-TDC due to jitter is $\sigma_{jitter}^{\left(n\right)}=\sqrt{2^{\left(n-1\right)}-1}\cdot \sigma_{jitter}$ [37].

Similar considerations apply to the effect of mismatch. The standard deviation of the total propagation time error of the *n*-bit SA-TDC due to mismatch is $\sigma_{mism}^{\left(n\right)}=\sqrt{2^{\left(n-1\right)}-1}\cdot \sigma_{mism}$ [37]. But differently from process mismatch, noise is a time-variant phenomenon, thus the timing deviation $\Delta t_{jitt,i}$ is different for each edge propagated through the *i*th delay element.

**Figure 39:**
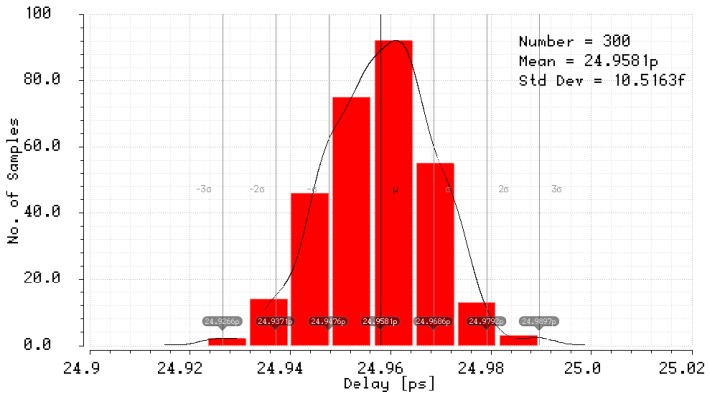
Simulation results of delay jitter for unit delay element (pair of inverters) for 180 nm CMOS process.

**Figure 40:**
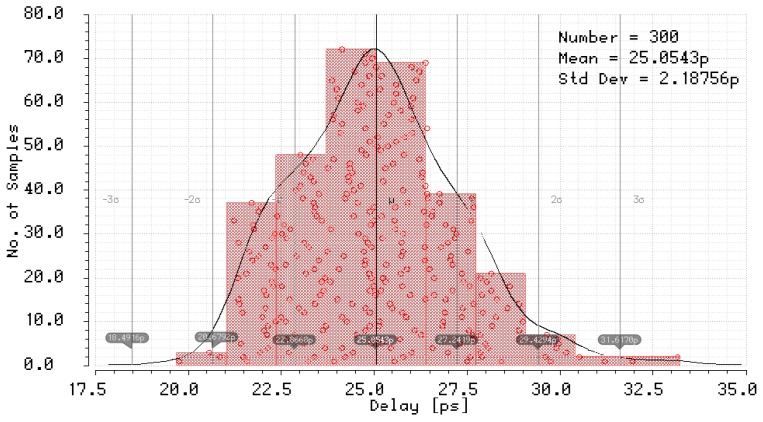
Simulation results of mismatch for unit delay element (pair of inverters) for 180 nm CMOS process.

The simulation results of the mismatch and jitter time for the pair of inverters in the standard 180 nm CMOS process with a nominal delay $T_{0}$ of about 25 ps give $\sigma_{jitter}$ and $\sigma_{mism}$ equal to 10.52 fs (Figure 39) and 2.19 ps (Figure 40), respectively. Both results for 8-bit SA-TDC correspond to $\sigma_{jitter}^{\left(8\right)}=\sqrt{127}\cdot 10.52=118.6$ fs, and $\sigma_{mism}^{\left(8\right)}=\sqrt{127}\cdot 2.19=24.7$ ps, which is slightly lower than *LSB*.

As follows from the above evaluations, the SA-TDC performance is degraded mostly by the mismatch in the fabrication process. The solutions to mitigate the error induced by the mismatch (i.e., delay offset between the tracks *S* and *R*, and the nonlinearity of the transfer function) have been presented in [32,33,47] and can be adopted in the proposed SA-TDC. The compensation of delay offset associated with on-die parameter variation can be achieved by a self-calibration scheme based on an on-chip calibration input timing generator [32]. Furthermore, the integral nonlinearity can be minimized by the use of a look-up-table with the measured INLs of the transfer function, or by linearization of delay lines with on-chip calibration structures [33,47].

### 7.6. Impact of Temperature and Supply Voltage Variations

**Figure 41:**
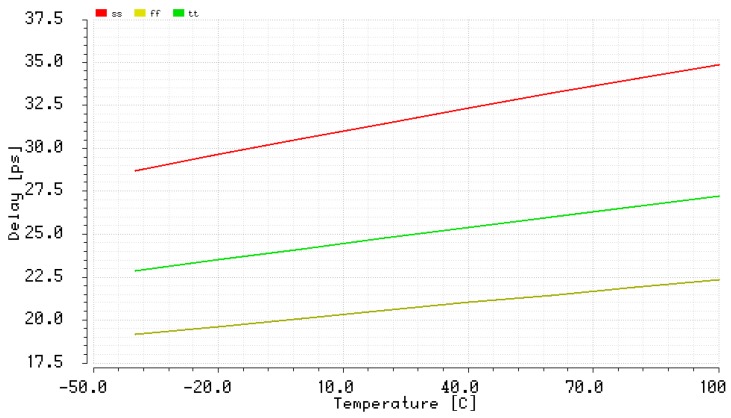
Unit delay vs. operating temperature for slow-slow (ss), typical-typical (tt) and fast-fast (ff) corners.

In order to evaluate the impact of temperature and supply voltage variations on SA-TDC performance, the designed unit delay has been simulated for slow-slow (ss), typical-typical (tt) and fast-fast (ff) corners between −30 °C and 100 °C. At the typical operation conditions and room temperature, the unit delay is around 24.98 ps while it is around 31.76 ps for the worst case based on the ss corner (Figure 41).

**Figure 42:**
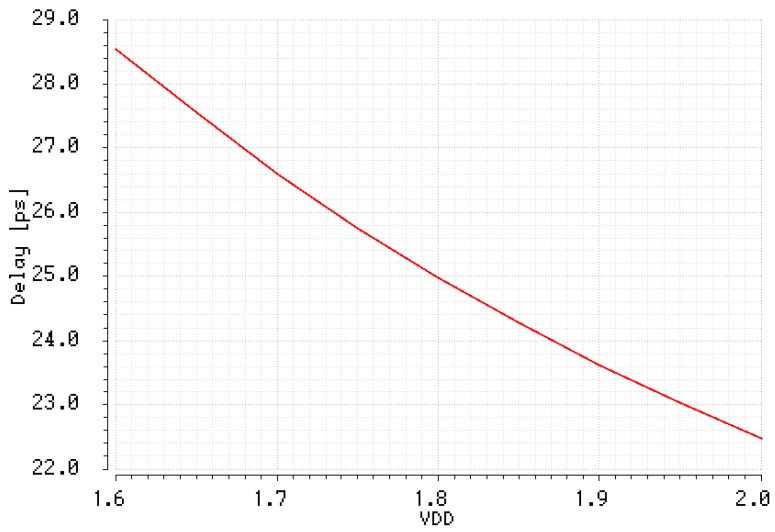
Unit delay vs. voltage supply.

The designed single pair of inverters operates at the nominal voltage supply (*V_DD_*) of 1.8 V and the delay of the device depends on the variability of the *V_DD_*. The impact of voltage supply variations on the unit delay is presented in Figure 42. The simulation experiment shows that the propagation delay varies from 22.47 ps to 28.54 ps if *V_DD_* varies between 1.6 V and 2.0 V. At higher voltage supply the pair of inverters operates faster at the price of increase of the power consumption.

## 8. Conclusions

In deep-submicron CMOS technologies, the resolution of encoding signals in the time domain becomes superior to the resolution in the voltage domain, which promotes representing information by the time intervals between discrete events rather than by the voltages, or currents in electric network. Time-to-digital converters are an enabler for the time-domain digital processing of continuous signals. This study gives a tutorial on successive approximation TDCs (SA-TDCs) in feedforward architecture on one hand, and makes the contribution to optimization of SA-TDC design on the other. The proposed SA-TDC optimization consists essentially in reduction of circuit complexity and die area, as well as in improving converter performance. The main design improvement presented in the paper is the concept of removing one of two sets of delay lines from the SA-TDC feedforward architecture at the price of simple output decoding. For 12 bits of resolution, the complexity reduction is close to 50%. Furthermore, the paper presents the implementation of 8-bit SA-TDC in 180 nm CMOS technology with a quantization step 25 ps obtained by asymmetrical design of pair of inverters and symmetrizing multiplexer control. Future research may address the implementation of SA-TDC with single set of delay lines in modern CMOS processes with finer time resolution.

## Figures and Tables

**Table 1:** Number of transistors in *n*-bit SA-TDC with two sets of delay lines, and with single set of delay lines and output decoding for *T_m_* = 0 and *T_m_* = 250 ps.

Number of Bits *n*	Basic SA-TDC with Two Sets of Delay Lines		SA-TDC with Single Set of Delay Lines and Output Decoding	
	*T_m_* = 0	*T_m_* = 250 ps	*T_m_* = 0	*T_m_* = 250 ps
2	60	140	68	148
3	116	276	128	288
4	188	428	196	436
5	292	612	280	600
6	460	860	396	796
7	756	1236	576	1056
8	1308	1868	884	1444
9	2372	3012	1448	2088
10	4460	5180	2524	3244
11	8596	9396	4624	5424
12	16,828	17,708	8772	9652

**Table 2:** Transistors dimensions for asymmetric pair of inverters.

Transistor	W (µm)	L (µm)	Fingers
P_1_	0.27	0.18	1
N_1_	12.22	0.18	47
P_2_	18.00	0.18	1
N_2_	0.27	0.18	1

**Table 3:** Parameters of SA-TDC with single set of delay lines and symmetrized multiplexers implemented in 180 nm CMOS technology.

Bits	±*T* (ps)	*T*_0_ (ps)	*T_m_* (ps)	RMS DNL	RMS INL
8	3175	25	250	0.14	0.16

**Table 4:** Comparison of this work to previous works on SA-TDCs.

Feature	Reference			
	[33]	[32]	[31]	This Work
**Architecture**	Feedback	Feedforward	Feedforward	Feedforward
**Technology (nm)**	350	65	65	180
**Nominal resolution (ps)**	1.22	9.77	50	25
**Number of bits**	13	10	5	8
**Full scale (ns)**	0.32	10	1.5	6.35
**Number of sets of binary-scaled delay components**	2	2	2	1
